# *Ajuba* as a Potential Nutrition-Responsive Biomarker for the Prevention of Age-Related Sarcopenia

**DOI:** 10.3390/ijms26167869

**Published:** 2025-08-14

**Authors:** Youngji Han, Seung Pil Pack

**Affiliations:** 1 Bio-Medical Research Institute, Kyungpook National University Hospital, Daegu 41940, Republic of Korea; youngjihan@knu.ac.kr; 2 Biological Clock-Based Anti-Aging Convergence RLRC, Korea University, Sejong-ro 2511, Sejong 30019, Republic of Korea; 3 Department of Biotechnology and Bioinformatics, Korea University, Sejong-ro 2511, Sejong 30019, Republic of Korea

**Keywords:** sarcopenia, nutrition-responsive biomarker, nutritional therapy, *Ajuba*

## Abstract

Sarcopenia, the age-related decline in skeletal muscle mass and function, is a growing health concern in aging populations. Nutritional interventions are increasingly recognized for their therapeutic potential; however, molecular biomarkers that reflect their efficacy are limited. To identify nutrition-responsive genes relevant to sarcopenia, we performed transcriptomic profiling of gastrocnemius muscle from mature and middle-aged mice. Aging-associated differentially expressed genes (DEGs) were filtered based on expression levels and correlation with muscle mass. Functional food interventions, including high- and low-molecular-weight collagen hydrolysates and allulose, were applied, and effect scores were calculated to assess transcriptomic responsiveness. *Ajuba*, a gene involved in cytoskeletal regulation and tissue remodeling, was significantly downregulated in middle-aged mice, consistent with aging-associated muscle decline. Dietary supplementation restored *Ajuba* expression across all intervention groups, with the strongest effect observed in the high-molecular-weight collagen group. *Ajuba* expression also showed strong positive correlations with tibialis anterior mass, hindlimb thickness, and muscle-to-fat ratio. *Ajuba* was identified as a nutritionally modifiable gene with strong associations to muscle phenotype and dietary response. These findings support *Ajuba* as a transcriptomic biomarker and potential molecular target for precision nutrition strategies aimed at preventing or mitigating sarcopenia.

## 1. Introduction

Sarcopenia, characterized by the progressive and age-related decline in skeletal muscle mass and function, is a major factor contributing to frailty, falls, metabolic disorders, and mortality in the elderly population [1,2]. With the global increase in aging populations, there is an urgent need to develop effective strategies to prevent or delay sarcopenia [3]. Among various non-pharmacological approaches, dietary intervention using functional food components has attracted attention as a relatively safe and sustainable method [4,5]. Nutritional compounds have been reported to improve muscle health by reducing inflammation, oxidative stress, and metabolic dysregulation [6,7].

However, the molecular mechanisms underlying the effects of these dietary components remain largely unclear. In particular, little is known about how they modulate gene expression and metabolic pathways in vivo. Moreover, it is uncertain whether such gene-level responses can serve as reliable biomarkers to guide early prevention. To develop effective dietary strategies, it is essential to identify molecular markers that reflect biological responsiveness to nutritional intervention [8].

Another important consideration is that the same dietary intervention may not produce uniform outcomes across individuals. This variability has led to increasing interest in personalized nutrition, which considers individual physiological and genetic characteristics [9,10]. Personalized nutrition seeks to optimize dietary approaches using biomarkers that indicate an individual’s responsiveness to specific foods or nutrients. Therefore, identifying genes that are both regulated by diet and associated with meaningful physiological changes is a critical goal in the field of precision nutrition [11,12].

As we previously reported, we examined the effects of molecular weight-specific collagen hydrolysates and allulose as dietary interventions for age-related sarcopenia [13,14]. Supplementation with collagen hydrolysates in middle-aged mice increased skeletal muscle mass, reduced adiposity, and improved muscle structure by activating the IGF-1/PI3K/AKT/mTOR signaling pathway [13]. Allulose also enhanced muscle mass and strength, potentially by upregulating IGF-1, suppressing myostatin, modulating autophagy via the mTOR pathway, and increasing antioxidant activity [14]. Building on these findings, the present study aimed to investigate how supplementation with these functional food components affects skeletal muscle gene expression in a mouse model of age-related sarcopenia. Through transcriptome analysis, we aimed to identify genes that were altered in response to dietary intervention and evaluate their potential as nutrition-responsive biomarkers. This approach may provide insight into the molecular mechanisms of sarcopenia and offer a basis for developing targeted nutritional strategies to support muscle health during aging.

## 2. Results

### 2.1. Identification of Muscle-Related Candidate Genes Among Aging-Associated DEGs

To investigate the molecular effects of dietary interventions on age-related sarcopenia, we utilized a mouse model involving four experimental groups. Eight-week-old male mice receiving a standard diet served as the mature control group (Mature), while 48-week-old male mice were assigned to either a middle-aged control group (Middle-aged; standard diet) or to dietary intervention groups receiving 3% allulose (ALL) or 0.2% collagen hydrolysates of low or high molecular weight (LCOL and HCOL). All interventions were conducted for 12 weeks. Each group was evaluated for phenotypic and transcriptomic changes, and individual mice were treated as independent experimental units. Although the mice were initially 8 and 48 weeks old at the time of group allocation, all animals underwent a one-week acclimation period followed by the 12-week intervention. Therefore, transcriptomic and phenotypic comparisons were effectively made between 21-week-old (formerly 8-week-old) and 61-week-old (formerly 48-week-old) mice. Detailed information regarding animal grouping and dietary composition is provided in Section 4.1.

To explore transcriptional changes associated with aging, we performed mRNA sequencing using gastrocnemius muscle tissues from all experimental groups. For the identification of aging-related differentially expressed genes (DEGs), we first focused on a comparison between the Mature and Middle-aged groups, both maintained on a standard diet without any dietary intervention. Based on a threshold of false discovery rate (FDR) < 0.05, a total of 1300 aging-related DEGs were identified (Supplementary Material Table S1). However, these DEGs may reflect general aging processes rather than being specifically related to age-associated sarcopenia.

**Figure 1:**
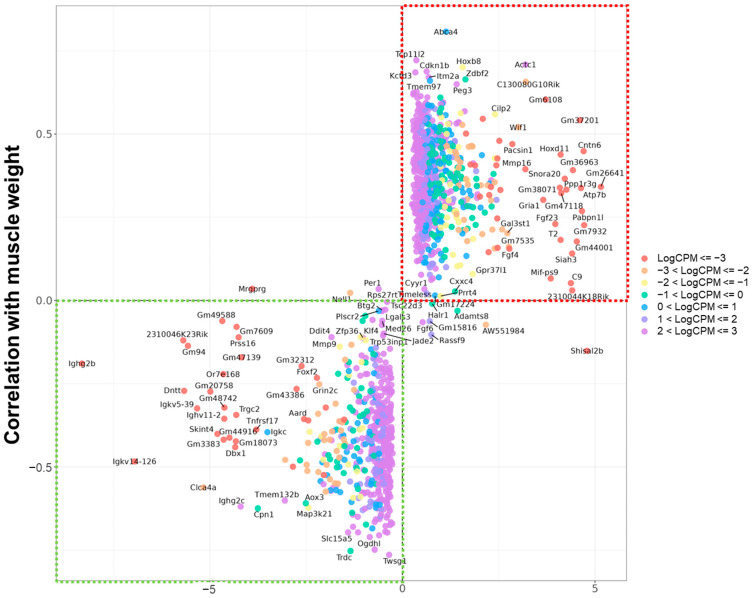
Scatter plot of age-related gene expression changes and correlation with total muscle weight. Genes are plotted according to their log fold change between middle-aged and mature mice (*x*-axis) and their correlation with total muscle mass per 100 g of body weight (*y*-axis). Genes in the upper right quadrant (red box) show increased expression with aging and positive correlation with muscle mass, while genes in the lower left quadrant (green box) show decreased expression and negative correlation, indicating possible involvement in sarcopenia-related mechanisms.

To refine this gene list and identify sarcopenia-relevant candidates, we assessed the correlation between gene expression levels and total muscle weight normalized to 100 g of body weight (Figure 1). Genes with inconsistent directions, *Per1*, *Shisal2b*, *AW551984*, and those located in the second and fourth quadrants of the LogFC vs. correlation plot, were considered less likely to be directly involved in sarcopenia.

**Figure 2:**
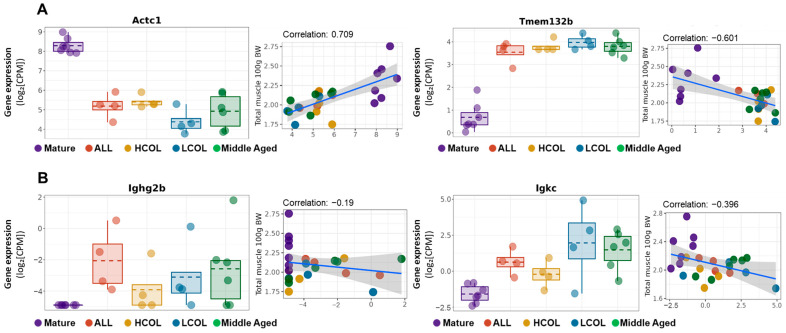
Expression and muscle weight correlation of aging-associated genes. (**A**) Genes showing consistent direction with total muscle mass and high expression levels (e.g., *Actc1*, *Trem132b*). (**B**) Genes showing consistent direction with total muscle mass but with low expression levels (e.g., *Ighg2b*, *Igkc*); Mature, 21 weeks old, *n* = 6; Middle aged, 61 weeks old, *n* = 6; ALL, allulose, 61 weeks old, *n* = 4; LCOL, low-molecular-weight collagen hydrolysate, 61 weeks old, *n* = 4; high-molecular-weight collagen hydrolysate groups, 61 weeks old, *n* = 4; Blue line represents the Pearson correlation regression line, and the gray shaded area indicates the 95% confidence interval.

Furthermore, although some DEGs exhibited large expression changes, many showed very low expression levels, with log-transformed counts per million (logCPM) values below −3, suggesting limited biological significance. However, several exceptions were identified. For instance, *Actc1* and *Tmem132b* demonstrated both high expression levels and a positive correlation with muscle mass, suggesting a potential functional role in muscle maintenance during aging (Figure 2A). Meanwhile, *Igkc* and *Ighg2b* also exhibited expression patterns consistent with sarcopenia progression; however, their overall expression levels were relatively low, and their correlations with muscle mass were weak (Figure 2B). Therefore, we restricted our candidate gene selection to those with LogCPM > 0 for further analysis to enhance biological relevance and biomarker potential.

### 2.2. Effect-Score-Based Prioritization of Allulose- and Collagen Hydrolysate-Responsive Aging Genes

To identify aging-related genes associated with sarcopenia that are responsive to each functional food intervention, we conducted an analysis based on effect scores. We first filtered DEGs that had logCPM > 0 and showed a correlation direction consistent with total muscle mass. Effect scores were then calculated within each intervention group (allulose, high-molecular-weight collagen (HCOL), and low-molecular-weight collagen (LCOL)). A lower effect score indicates a greater reversal of aging-related expression changes induced by the intervention.

**Figure 3:**
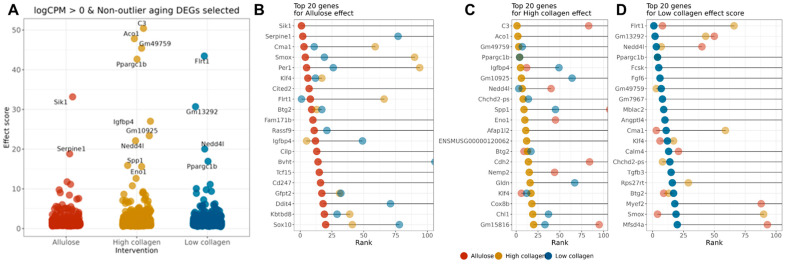
Effect-score-based prioritization of aging-related DEGs responsive to allulose and collagen hydrolysate interventions. (**A**) Distribution of effect scores for aging-related DEGs (logCPM > 0) showing consistent direction with muscle mass correlation, across three intervention groups: allulose (red), high-molecular-weight collagen (yellow), and low-molecular-weight collagen (blue). A higher effect score indicates a stronger reversal of age-associated gene expression changes by the intervention; (**B**–**D**) top 20 aging-related DEGs ranked by effect scores for each intervention group: (**B**) allulose, (**C**) high-molecular-weight collagen hydrolysate, and (**D**) low-molecular-weight collagen hydrolysate. Dot colors indicate the intervention group, and the horizontal axis represents the relative rank of the effect score (lower rank = more responsive). Commonly ranked genes across multiple groups, such as *Ppargc1b*, *Klf4*, and *Nedd4l*, may represent shared targets of dietary interventions, whereas group-specific genes (e.g., *Sik1*, *C3*, *Flrt1*) highlight intervention-specific effects.

As shown in Figure 3A, *Sik1* and *Serpine1* exhibited the highest effect scores in the allulose group, indicating high responsiveness to the intervention. In the HCOL group, *C3*, *Aco1*, *Gm49759*, and *Ppargc1b* showed strong effects, while in the LCOL group, *Flrt1*, *Gm13292*, and *Ppargc1b* ranked among the top genes.

Additionally, visualizing the top 20 genes by effect score in each intervention group (Figure 3B–D) revealed that *Klf4* and *Btg2* consistently appeared across all groups, highlighting their potential as a common target of intervention. In contrast, *Ppargc1b* was ranked among the top genes only in the collagen-supplemented groups, while *Sik1* and *C3* showed strong responses in specific groups, suggesting their utility as group-specific biomarkers.

### 2.3. Ajuba Identified as a Nutrition-Responsive Biomarker Downstream of Klf4

Among the top-ranked genes responsive to the interventions, *Klf4* was consistently identified across all groups.

**Figure 4:**
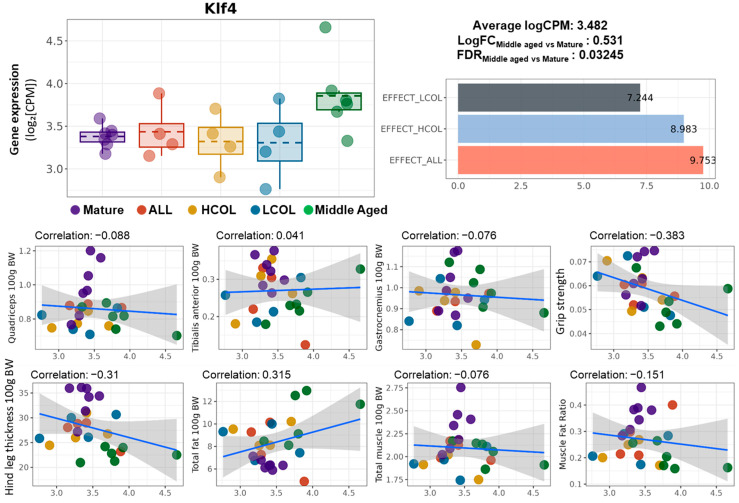
Expression of Klf4, intervention-specific effect scores, and correlation with muscle phenotypes. Boxplot of *Klf4* expression (logCPM) across experimental groups; summary statistics indicate the average expression level (logCPM = 3.482), log fold change between Middle-aged and Mature groups (LogFC = 0.531), and false discovery rate (FDR = 0.03245); LogFC values are calculated based on logCPM following normalization, representing the fold change in expression between Middle-aged and Mature groups; the bar graph shows effect scores of *Klf4* across interventions LCOL = 7.444, HCOL = 8.983, and ALL = 9.753; scatter plots show the Pearson correlation between *Klf4* expression and muscle-related phenotypes: quadriceps weight, tibialis anterior weight, gastrocnemius weight, grip strength, hindlimb thickness, total fat, total muscle, and muscle–fat ratio (all normalized to body weight); linear regression lines with 95% confidence intervals are shown with correlation coefficients indicated in each panel; Mature, 21 weeks old, *n* = 6; Middle aged, 61 weeks old, *n* = 6; ALL, allulose, 61 weeks old, *n* = 4; LCOL, low-molecular-weight collagen hydrolysate, 61 weeks old, *n* = 4; high-molecular-weight collagen hydrolysate groups, 61 weeks old, *n* = 4; Blue line represents the Pearson correlation regression line, and the gray shaded area indicates the 95% confidence interval.

To further investigate the role of *Klf4* as a nutrition-responsive biomarker, we examined its expression patterns and correlations with phenotypic features. Klf4 expression was significantly upregulated in aged mice (Middle vs. Mature, LogFC = 0.531, FDR = 0.03245), while its expression was partially restored by all three interventions (Figure 4). Effect score analysis revealed moderate recovery in the LCOL (7.44), HCOL (8.98), and allulose (9.75) groups, indicating *Klf4* responsiveness to dietary modulation.

However, the correlation between *Klf4* expression and muscle-related traits was generally weak. Except for total muscle mass (r = 0.407), most muscle indicators showed no strong association. In fact, negative correlations were observed with grip strength (r = −0.383) and hindlimb thickness (r = −0.310), suggesting that *Klf4* may not be directly linked to muscle mass itself but rather associated with broader changes in body composition or systemic metabolic states.

**Figure 5:**
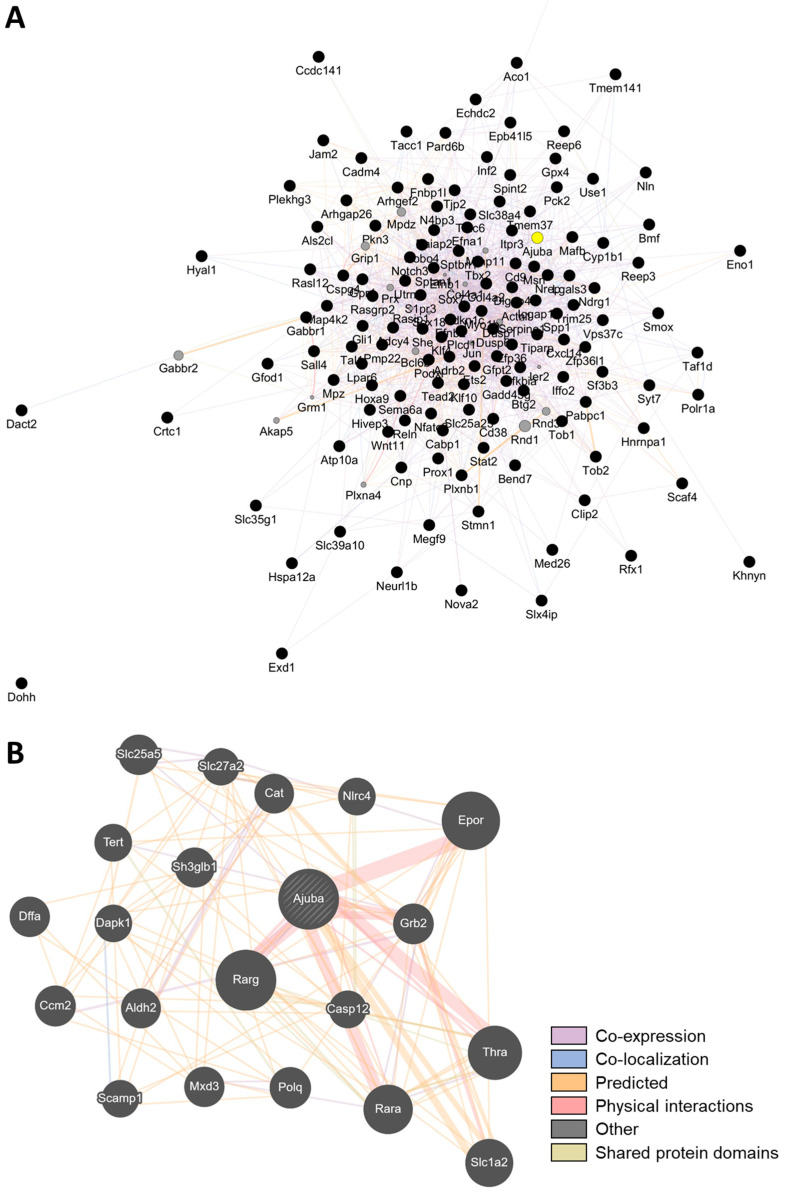
Network-based identification of Ajuba as a central KLF4-associated gene responsive to dietary interventions. (**A**) Network of *Klf4*-associated genes filtered for relevance to age-related sarcopenia (logCPM > 0) and responsiveness to functional food interventions (effect score > 1). The gene network was constructed using GeneMANIA based on the filtered gene set. (**B**) Subnetwork highlighting Ajuba, which acts as a central hub gene with connections across multiple interaction types, including co-expression, co-localization, predicted functional associations, and direct physical interactions. Diagonal hatching indicates that the gene belongs to multiple functional categories. The prominence of *Ajuba* in multiple data layers supports its integrative regulatory role in sarcopenia-related molecular pathways.

Building upon these findings, we further investigated downstream and co-regulated genes of Klf4 to identify functional mediators that may be nutritionally or genetically actionable. To refine sarcopenia-relevant biomarkers regulated by *Klf4*, we cross-referenced the *Klf4* target genes based on the ChEA (ChIP-X Enrichment Analysis) Transcription Factor Targets dataset provided by Harmonizome 3.0 [15], with muscle-related candidate genes identified from aging-associated differentially expressed genes (DEGs) in the first step of our analysis. Among the KLF4-associated genes filtered for age-related sarcopenia relevance and responsiveness to functional food interventions (logCPM > 0, effect score > 1), we performed a GeneMANIA-based network analysis (Figure 5A). This revealed that *Ajuba* emerges as a highly interconnected node, demonstrating associations across multiple relationship categories—including co-expression, co-localization, predicted functional interactions, and direct physical interactions (Figure 5B). These results support *Ajuba* as a network-centered gene with potential integrative regulatory functions in sarcopenia-related pathways.

Notably, Ajuba emerged as a candidate of particular interest from this network-centered approach, showing both strong correlation with muscle traits and inclusion among the Klf4 targets identified by ChEA. Furthermore, Ajuba was among the genes with an effect score ≥ 1 in response to allulose, LCOL, and HCOL interventions, highlighting its potential as a nutritionally responsive biomarker for sarcopenia.

**Figure 6:**
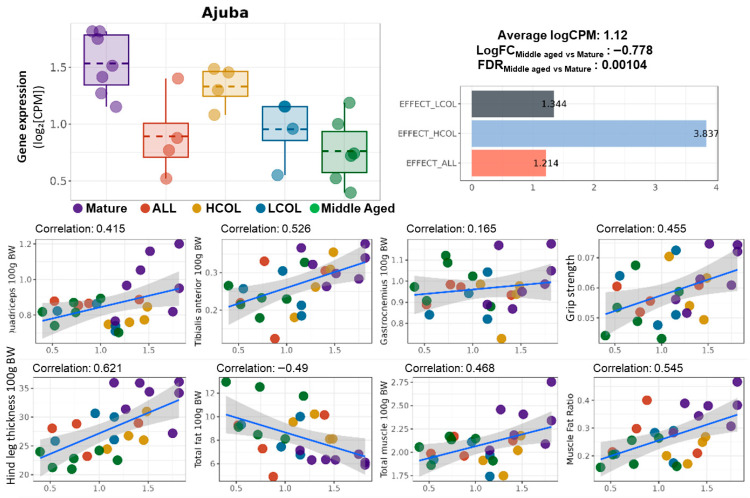
Expression of *Ajuba*, intervention-specific effect scores, and correlation with phenotypes. Boxplot of *Ajuba* expression (logCPM) across experimental groups; summary statistics indicate the average expression level (logCPM = 1.12), log fold change between Middle-aged and Mature groups (LogFC = –0.778), and false discovery rate (FDR = 0.00104); the bar graph shows the effect scores of *Ajuba* across interventions: LCOL = 1.344, HCOL = 3.837, and ALL = 1.214; LogFC values are calculated based on logCPM following normalization, representing the fold change in expression between Middle-aged and Mature groups; scatter plots show the Pearson correlation between *Ajuba* expression and muscle-related phenotypes: quadriceps weight, tibialis anterior weight, gastrocnemius weight, grip strength, hindlimb thickness, total fat, total muscle, and muscle–fat ratio (all normalized to body weight); linear regression lines with 95% confidence intervals are shown; correlation coefficients are indicated in each panel; Mature, 21 weeks old, *n* = 6; Middle aged, 61 weeks old, *n* = 6; ALL, allulose, 61 weeks old, *n* = 4; LCOL, low-molecular-weight collagen hydrolysate, 61 weeks old, *n* = 4; high-molecular-weight collagen hydrolysate groups, 61 weeks old, *n* = 4; Blue line represents the Pearson correlation regression line, and the gray shaded area indicates the 95% confidence interval.

This strengthens the rationale for *Ajuba* as a potential downstream effector of *Klf4*-mediated transcriptional regulation. *Ajuba* encodes an LIM-domain-containing protein implicated in cell cycle regulation, muscle repair, and Hippo signaling [16]. Although *Ajuba* was not among the top-ranked DEGs based solely on effect score, its biological relevance and connectivity to intervention-responsive transcriptional regulators such as *Klf4* warrant further investigation, as shown in Figure 6.

*Ajuba* expression was significantly decreased in middle-aged mice compared to mature controls (logFC = −0.778, FDR = 0.00104), indicating age-related transcriptional suppression (Figure 1). All three dietary interventions, including HCOL, LCOL, and allulose, increased *Ajuba* expression compared to untreated middle-aged mice. This suggests that these functional food ingredients contributed to reversing the aging-associated decline. Among the interventions, the HCOL group showed the greatest effect with a reversal index of 3.837, followed by LCOL (1.344) and allulose (1.214). These findings indicate that *Ajuba* is a nutritionally responsive gene whose expression can be modulated by various dietary components.

In addition, *Ajuba* expression levels were positively correlated with multiple muscle-related parameters, including tibialis anterior mass (correlation coefficient: 0.526), hind leg thickness (0.621), and muscle-to-fat ratio (0.545).

## 3. Discussion

In recent years, the scope of nutritional therapy has expanded beyond managing chronic diseases to encompass the prevention and treatment of age-related conditions [17,18]. Among these, sarcopenia, which is characterized by the progressive decline in muscle mass and function, poses a significant threat to the health and quality of life of the aging population, underscoring the urgent need for effective preventive and therapeutic strategies [19]. Dietary intervention, as a relatively safe and sustainable non-pharmacological approach, has gained considerable attention [20,21]. However, identifying reliable biomarkers that reflect responsiveness to dietary modulation remains a critical prerequisite for developing precise nutritional strategies.

This study aimed to identify gene-level biomarkers that reflect dietary responsiveness in the context of age-related sarcopenia. *Klf4* was initially observed to be significantly upregulated in aging skeletal muscle, consistent with its known involvement in cellular stress responses, inflammation, and cell cycle [22,23,24]. Interestingly, in our study, *Klf4* expression was higher in the Middle-aged group compared to the Mature group. Given that *Klf4* is known to suppress proliferation in various tissues, this age-related decline in *Klf4* expression may suggest impaired muscle regeneration in advanced aging [25,26]. However, our correlation analyses revealed that *Klf4* expression showed only weak or negative associations with muscle-related phenotypes, including tibialis anterior mass, hindlimb thickness, and grip strength. This indicates that *Klf4* upregulation in aged muscle may be indicative of broader transcriptional stress responses rather than direct involvement in maintaining muscle integrity.

Nevertheless, *Klf4* emerged as one of the most significantly modulated transcription factors in response to dietary interventions. Previous studies have suggested that *Klf4* may contribute to muscle differentiation and regeneration through target genes such as *p57* and *Myomixer* [27]. Based on this, we hypothesized that its downstream targets might mediate functionally relevant effects. Through co-expression and network-based analysis, we identified *Ajuba* as a downstream gene potentially regulated by *Klf4*. Notably, *Ajuba* expression was negatively associated with age and positively correlated with muscle-related phenotypes. These findings support the possibility that *Ajuba* may serve as a nutrition-responsive gene involved in early sarcopenic changes, mediated at least in part through transcriptional regulation by *Klf4*. This approach underscores the importance of transcription-factor-driven network analysis in uncovering molecular mediators of nutritional intervention. To identify more functionally relevant markers, we performed transcriptome-level screening and network-based analyses, which led to the identification of *Ajuba* as a promising gene of interest. *Ajuba* encodes an LIM-domain protein with critical roles in cytoskeletal organization, cell adhesion, tissue regeneration, and mechanical signal transduction [28]. Importantly, *Ajuba* functions as a scaffold for the Hippo signaling pathway, where it regulates LATS1/2 kinase activity and modulates the subcellular localization of YAP/TAZ, transcriptional regulators involved in growth and regeneration [29]. In our aging model, *Ajuba* expression was markedly downregulated in middle-aged mice, and this reduction was reversed by all three dietary interventions: high- and low-molecular-weight collagen and allulose.

Recent studies have further elucidated the functional role of *Ajuba* in cellular architecture [30,31]. Specifically, *Ajuba* has been shown to participate in a Rac–PAK1–*Ajuba* feedback loop that regulates actin remodeling and reinforces cadherin-mediated cell–cell adhesion [32]. Upon cell contact, PAK1 transiently phosphorylates *Ajuba* at Thr172, enhancing its interaction with active Rac and promoting localized actin polymerization [32]. This interaction is essential for stabilizing adherens junctions and maintaining mechanical resilience under stress. Rather than simply serving as a downstream effector, *Ajuba* dynamically modulates Rac signaling at junctional sites, integrating mechanical cues with cytoskeletal reorganization [33]. Given these mechanistic insights, the restoration of *Ajuba* expression in response to dietary interventions may reflect not only transcriptional recovery but also the reactivation of cellular pathways critical for structural adaptation and muscle integrity in aging tissue.

From a translational standpoint, these findings support the incorporation of tissue-specific molecular readouts when evaluating dietary interventions. Whereas traditional biomarkers focus on systemic metabolic or inflammatory parameters, Ajuba offers a direct readout of musculoskeletal adaptation. In our study, Ajuba’s effect score increased to 3.84 with HCOL, 1.34 with LCOL, and 1.21 with allulose. These robust responses highlight *Ajuba*’s potential as a biomarker for assessing the efficacy of sarcopenia-targeted nutritional strategies. Nonetheless, several limitations should be considered. This study was conducted in a murine model, which has a limited sample size, potentially affecting the generalizability of the results to human populations. In addition, the group sizes were relatively small and uneven, which may introduce statistical bias and reduce the power to detect subtle effects. Moreover, only male mice were used, limiting the applicability of the findings across sexes. Also, in this study, dietary interventions were exclusively applied to middle-aged mice to evaluate their effectiveness in mitigating age-related sarcopenia. The young mice served as a physiological reference group representing optimal muscle condition and therefore did not receive any dietary intervention. While dietary effects in younger animals may be of interest, exploring such effects was beyond the scope of this study and not aligned with its primary aim. Furthermore, protein-level validation of *Ajuba* expression and localization was not performed, and functional studies such as gene knockdown or overexpression are necessary to clarify its causal role. Future research should also explore whether epigenetic or chromatin-level changes contribute to the regulation of *Ajuba* expression in response to aging and dietary intervention.

## 4. Materials and Methods

### 4.1. Animals and Diet

A total of 21 male C57BL/6J mice (48 weeks old) and 7 male C57BL/6J mice (8 weeks old) were obtained from JA BIO (Suwon, Republic of Korea). After a one-week acclimation period with commercial chow, the 48-week-old mice were randomly allocated into three dietary intervention groups: (1) middle-aged control (Middle aged, fed AIN-93G diet, *n* = 6), (2) allulose (ALL, 3% allulose replacing cellulose in AIN-93G, *n* = 4), and (3) 0.2% low- and high-molecular-weight collagen hydrolysate groups (LCOL and HCOL, *n* = 4, respectively). The 8-week-old mice served as the mature control group (Mature), receiving the standard AIN-93G diet from the 8th week onward (Table 1). Thus, a total of five experimental groups were compared: Mature (control), Middle (age-matched untreated), ALL, LCOL, and HCOL. All dietary interventions were administered for 12 weeks. At the end of the intervention, the mature control group reached 21 weeks of age, while all other groups were 61 weeks old.

Each individual mouse was treated as a single experimental unit for all analyses, including phenotyping and transcriptomic assessment. All mice were housed under identical environmental conditions (20–23 °C, with alternating 12 h light/dark periods) and provided ad libitum access to food and water. Cages were arranged randomly within the animal facility and rotated weekly to avoid location-specific bias. Phenotypic assessments were performed in a randomized order by an investigator who was blinded to the group allocation. Body weight, food intake, and blood glucose were monitored regularly throughout the experimental period. All procedures were approved by the Institutional Animal Care and Use Committee of Kyungpook National University (Approval No. KNU-2020-109). Phenotypic improvements related to sarcopenia have been previously reported [13,14]. The current study focuses on transcriptomic changes in skeletal muscle tissues collected from the same experimental groups.

### 4.2. mRNA-Seq and Analysis

Total RNA was extracted from gastrocnemius tissue, and libraries were prepared using the TruSeq Stranded mRNA kit. Sequencing was performed on the Illumina NextSeq 500 (paired-end 2 × 75 bp, Macrogen, Republic of Korea). Quality control was performed using FastQC v0.11.9 [34] and Trimmomatic v0.39 [35], and reads were aligned to the mouse reference genome using STAR (v2.6.0c) [36]. Gene expression was quantified using StringTie (v2.1.3b) [37]. Gene annotations were retrieved from the Ensembl database using the biomaRt R package (2.62.0), accessing Ensembl release 75 [38]. Differential gene expression analysis across dietary intervention groups and age groups was conducted using generalized linear models (GLMs) within the edgeR package [39]. Genes with a false discovery rate (FDR) < 0.05 and log-CPM > 0 were considered differentially expressed. The complete RNA-seq dataset, including raw and processed files, is publicly available in the NCBI Gene Expression Omnibus (GEO) under accession number GSE175562.

### 4.3. Effect Score Calculation

To evaluate the responsiveness of aging-related genes to each dietary intervention, we defined an effect score based on the magnitude of reversal in age-associated changes in gene expression. First, differentially expressed genes (DEGs) were identified between mature and middle-aged mice (Middle vs. Mature), and those with consistent correlation direction with total muscle mass and a mean expression level greater than logCPM > 0 were selected.

For each intervention group (allulose, HCOL, LCOL), the effect score was calculated as follows:

$$Effect\;Score=\frac{|\;{LogFC}_{Middle\;aged\;vs.\;Mature}\;|}{|\;{LogFC}_{Intervention\;vs.\;Middle\;aged}\;|}$$

A higher effect score indicates a greater reversal of aging-related expression changes by the intervention. Genes with low effect scores were considered less responsive to intervention. The effect scores were computed individually for each gene and intervention group using the absolute values of log fold changes obtained from differential expression analysis.

### 4.4. WGCNA and Integrated Analysis

Weighted Gene Co-expression Network Analysis (WGCNA) was applied to the transcriptomic datasets to identify coordinated gene modules with sarcopenic traits [40]. The integrated analysis of transcriptomic data was conducted in the RStudio environment (R version 4.2.0) using various bioinformatics packages, including WGCNA, edgeR, tidyverse, ggplot2, ggpubr, doParallel, and foreach. Module–trait relationships were computed using Pearson correlation to explore associations between eigengene values and phenotypic measures.

Eight muscle-related phenotypes were evaluated, encompassing both morphological and functional indicators of sarcopenia. Morphological metrics included quadriceps, tibialis anterior, and gastrocnemius muscle weights, hindlimb thickness, total muscle mass, total fat mass, and muscle-to-fat ratio. Functional performance was assessed using grip strength, measured as the average of three trials using a calibrated grip strength meter. All values were normalized to 100 g of body weight. Hindlimb thickness was measured at week 12 to reflect treatment effects.

### 4.5. Gene Interaction Network Analysis

To investigate the functional relationships among candidate genes responsive to dietary interventions, we conducted gene interaction network analysis using GeneMANIA (https://genemania.org/, accessed on 12 August 2025) [41]. The analysis included genes with logCPM > 0 and effect scores > 1 across the intervention groups. GeneMANIA integrates various types of functional data, such as co-expression, physical interactions, co-localization, and predicted associations, to construct biologically relevant gene networks.

## 5. Conclusions

We propose *Ajuba* as a nutrition-responsive molecular biomarker that reflects dietary intervention-induced improvements in skeletal muscle aging. Although *Klf4* was consistently identified as an upstream regulator responsive to all interventions, its weak association with muscle phenotypes limited its utility as a direct biomarker. Through network-based analysis, *Ajuba* emerged as a downstream effector of *Klf4* with strong correlations to muscle traits and consistent responsiveness across all dietary groups. These findings support *Ajuba* as a promising molecular target for precision nutrition strategies aimed at preventing or delaying sarcopenia.

## Figures and Tables

**Table 1:** Diet composition for animal experiment.

Ingredient (g)	AIN-93G	AIN-93G + 3% ALL	AIN-93G + 0.2% LCOL	AIN-93G + 0.2% HCOL
Casein	200	200	200	200
Corn starch	397.5	397.5	397.5	397.5
Sucrose	100	100	100	100
Maltodextrin	132	132	132	132
Cellulose	50	20	48	48
Soybean oil	70	70	70	70
Mineral mix *	35	35	35	35
Vitamin mix ^†^	10	10	10	10
TBHQ, antioxidant	0.014	0.014	0.014	0.014
L-Cystine	3	3	3	3
Choline bitartrate	2.5	2.5	2.5	2.5
Allulose		30		
Low-molecular-weight collagen hydrolysate	-		2	-
High-molecular-weight collagen hydrolysate	-		-	2
Total (g)	1000	1000	1000	1000
Total protein contents (g)	200	200	202	202
Calories (cal/g)	3948	3948	3956	3956

The Mature group and the Middle-aged group were both maintained on the standard AIN-93G diet. LCOL, low-molecular-weight collagen; HCOL, high-molecular-weight collagen; ALL, allulose. * Mineral Mixture (AIN-93G) (gram/kg): Calcium Carbonate 357, Monopotassium Phosphate 196%, Potassium Citrate Monohydrate 70.78, Sodium Chloride 74, Potassium Sulfate 46.6, Magnesium Oxide 24, Ferric Citrate 6.06, Zinc Carbonate 1.65, Manganese Carbonate 0.63, Copper Carbonate 0.3, Potassium Iodate 0.01%, Sodium Selenate, Anhydrous 0.0103, Ammonium Molybdate 4H_2_O 0.00795, Sodium Metasilicate 9H_2_O 1.45, Chromium Potassium Sulfate 12H_2_O 0.275, Lithium Chloride 0.0174, Boric Acid 0.08145, Sodium Fluoride 0.0635, Nickel Carbonate 0.0318, Ammonium Vanadate 0.0066, Powdered Sugar 221. ^†^ Vitamin mix (AIN-93G) (gram/kg): Nicotinic Acid 3.00, D-Calcium Pantothenate 1.60, Pyridoxine HCl 0.70, Thiamine HCl 0.60, Riboflavin 0.60, Folic Acid 0.20, D-Biotin 0.02, Vitamin B12 (0.1% triturated in mannitol) 2.50, a-Tocopherol Powder (250 U/gm) 30.00, Vitamin A Palmitate (250,000 U/gm) 1.60, Vitamin D3 (400,000 U/gm) 0.25, Phylloquinone 0.075, Powdered Sucrose 959.655.

## Data Availability

The datasets used are available on request from the authors.

## Supplementary Materials

The following supporting information can be downloaded at https://www.mdpi.com/article/10.3390/ijms26167869/s1.
